# Supplementary material for: The gender dimensions of mental health during the Covid-19 pandemic: A path analysis
Source: PLoS One. 2023 May 19;18(5):e0283514. doi: 10.1371/journal.pone.0283514 (PMC10198511; doi:10.1371/journal.pone.0283514)
Supplement: S1 Table — (DOCX) [file pone.0283514.s001.docx]

**S1 Table. Missing data on analysis variables.**

| **Variable** | **Missing – Frequency (%)** |
| --- | --- |
| Age | 0 |
| GHQ (Pre-pandemic) | 95 (0.9) |
| GHQ (May) | 82 (0.8) |
| GHQ (July) | 128 (1.3) |
| Loneliness | 18 (0.2) |
| Hardly ever or never |  |
| Some of the time |  |
| Often |  |
| Hours of childcare | 149 (1.5) |
| Hours of housework | 287 (2.9) |
| Employment Disruption | 43 (0.4) |
| Household Income Quintiles | 253 (2.6) |
| Very High |  |
| High |  |
| Middle |  |
| Low |  |
| Very Low |  |
