# Supplementary material for: The gender dimensions of mental health during the Covid-19 pandemic: A path analysis
Source: PLoS One. 2023 May 19;18(5):e0283514. doi: 10.1371/journal.pone.0283514 (PMC10198511; doi:10.1371/journal.pone.0283514)
Supplement: S2 Table — (DOCX) [file pone.0283514.s002.docx]

**S2 Table. Distribution of sample characteristics among complete cases and cases with missingness on exogenous variables.**

| **Variable** | **Complete Cases (n=9,351)** | **Missing on exogenous variables (n=343)** |
| --- | --- | --- |
| Female – n/% | 5,439 (58.2) | 216 (62.97) |
| Age – mean/SD | 54.7 (15.5) | 49.3 (17.3) |
| Country – n/% |  |  |
| England | 7,639 (81.7) | 266 (77.6) |
| Scotland | 794 (8.5) | 33 (9.6) |
| Wales | 559 (5.9) | 19 (5.5) |
| Northern Ireland | 359 (3.8) | 25 (7.3) |
| GHQ (Pre-pandemic) – mean/SD | 11.06 (5.3) | 11.8 (5.6) |
| GHQ (May) – mean/SD | 12.02 (5.7) | 12.9 (6.7) |
| GHQ (July) – mean/SD | 11.4 (5.5) | 12.5 (6.6) |
| Loneliness – n/% |  |  |
| Hardly ever or never | 6,231 (66.8) | 199 (58.4) |
| Some of the time | 2,513 (26.9) | 115 (33.7) |
| Often | 591 (6.3) | 27 (7.9) |
| Hours of childcare – mean/SD | 3.7 (11.7) | 4.2 (12.7) |
| Hours of housework – mean/SD | 12.5 (8.7) | 12.8 (8.9) |
| Employment Disruption – n/% | 2,054 (22.06) | 92 (26.9) |
| Household Income Quantiles – mean/SD |  |  |
| Very High | 4,058.3 (1694.1) | 3,878.02 (1059.8) |
| High | 2,478.7 (198.6) | 2,534.03 (221.8) |
| Middle | 1,925.5 (136.7) | 1,927.7 (142.4) |
| Low | 1491.5 (120.2) | 1488.7 (104.4) |
| Very Low | 917.06 (314.6) | 939.53 (209.4) |
| Ethnicity – n/% |  |  |
| White British/Irish | 8,290 (88.7) | 274 (79.9) |
| Other White background | 282 (3.02) | 6 (1.8) |
| Mixed background | 133 (1.4) | 3 (0.9) |
| Indian | 206 (2.2) | 25 (7.3) |
| Pakistani | 109 (1.2) | 14 (4.1) |
| Bangladeshi | 40 (0.4) | 6 (1.8) |
| Black Caribbean | 73 (0.8) | 3 (0.9) |
| Black African | 53 (0.6) | 6 (1.8) |
| Other Non-White Background | 165 (1.8) | 6 (1.8) |
| Living with a partner – n/% | 5,439 (58.2) | 216 (62.9) |
