# Supplementary material for: The gender dimensions of mental health during the Covid-19 pandemic: A path analysis
Source: PLoS One. 2023 May 19;18(5):e0283514. doi: 10.1371/journal.pone.0283514 (PMC10198511; doi:10.1371/journal.pone.0283514)
Supplement: S3 Table — (DOCX) [file pone.0283514.s003.docx]

**S3 Table. Linear regression model results for the relationship between gender and GHQ in May and July.**

| **Model** | **Unadjusted** | | **Adjusted for age, household income quintiles, and GHQ in 2019** | |
| --- | --- | --- | --- | --- |
|  | Coefficient (95% CI) | P-value | Coefficient (95% CI) | P-value |
| **May GHQ** | 1.42 (0.95, 1.8) | <0.001 | 0.74 (0.34, 1.15) | <0.001 |
| **July GHQ** | 1.1 (0.63, 1.59) | <0.001 | 0.45 (0.025, 0.88) | 0.038 |
