# Supplementary material for: The gender dimensions of mental health during the Covid-19 pandemic: A path analysis
Source: PLoS One. 2023 May 19;18(5):e0283514. doi: 10.1371/journal.pone.0283514 (PMC10198511; doi:10.1371/journal.pone.0283514)
Supplement: S4 Table — (DOCX) [file pone.0283514.s004.docx]

**S4 Table. Fit indices for the mediation models for gender and mental health in May and July.**

| **Model** | **RMSEA** | **CFI** | **TLI** |
| --- | --- | --- | --- |
| **May** | 0.044 | 0.92 | 0.72 |
| **July** | 0.047 | 0.91 | 0.69 |
